# Functioning Problems in Persons with Schizophrenia in the Russian Context

**DOI:** 10.3390/ijerph181910276

**Published:** 2021-09-29

**Authors:** Manuel Rojas, Maite Barrios, Juana Gómez-Benito, Nadezhda Mikheenkova, Sergey Mosolov

**Affiliations:** 1 Department of Social Psychology and Quantitative Psychology, Faculty of Psychology, University of Barcelona, Passeig de la Vall d’Hebron, 171, 08035 Barcelona, Spain; mrojasc@ub.edu (M.R.); juanagomez@ub.edu (J.G.-B.); 2 Group on Measurement Invariance and Analysis of Change (GEIMAC), Institute of Neuroscience, Passeig de la Vall d’Hebron, 171, 08035 Barcelona, Spain; 3 Moscow Research Institute of Psychiatry, Poteshnaya ul., 3, 107076 Moscow, Russia; nadimixx@gmail.com (N.M.); profmosolov@mail.ru (S.M.); 4 Russian Medical Academy of Continuous Professional Education, Barrikadnaya pl., 2/1, 125993 Moscow, Russia

**Keywords:** ICF Core Set, schizophrenia, Russia, functioning

## Abstract

Assessing functionality in schizophrenia from a biopsychosocial perspective is essential to generate treatments that respond to the needs of the individual in his/her context. This research aims to assess the prevalence of functioning problems and their association with socio-demographic and clinical variables in a sample of Russian individuals with schizophrenia, using the International Classification of Functioning, Disability, and Health as a framework. An empirical cross-sectional study assessed the functioning of 40 individuals with schizophrenia using the International Classification of Functioning, Disability, and Health Core Set for schizophrenia. For the *Body functions* component, the highest prevalence of problems was found in *b144 Memory functions* (75%) and *b140 Attention functions* (70%). In the *Activities and participation* component, the greatest limitations were in *d770 Intimate relationships* (79.3%) and *d240 Handling stress and other psychological demands* (82.5%). In the *Environmental factors*, the most frequent problems were in *e110 Products or substances for personal consumption* (25%) and *e460 Societal attitudes* (22.5%); when scored as facilitators, the highest rated categories were *e125 Products and technology for communication* (100%) and *e165 Assets* (100%). These results may guide the design of specific treatments for these individuals and serve as a starting point for further studies in similar contexts and in other regions in Russia.

## 1. Introduction

Schizophrenia is a chronic mental illness characterized by significant impairments in cognitive and affective processes. The most common symptoms include positive and negative symptoms such as a sense of disconnection with reality, hallucinations, delusions, thought disorder, social withdrawal, difficulty showing emotions, disruptive or abnormal motor behavior, and difficulty functioning normally [1]. Currently, 23 million people around the world suffer from schizophrenia [2] and according to the statistical report of the Ministry of Public Health of the Russian Federation in 2017 the prevalence of schizophrenia in Russia was 0.354% or 520,356 people, and the prevalence of patients aged 20–59 years was 281,371 people (0.192%) [3]. Additionally, due to the progressive clinical deterioration it entails, schizophrenia is one of the 22 leading causes of major disability in people aged between 25 and 49 in terms of disability-adjusted life years (DALYs) [4]. In Russia every fourth patient with schizophrenia has a disability, and among all mentally ill people, 33.3 % are patients with schizophrenia [3].

Despite the high impact of schizophrenia on psychosocial adjustment, comprehensive evaluations of the functioning of these individuals are lacking. The use of specific measures to evaluate functioning in people with schizophrenia can provide relevant information on the interaction of personal, illness-related, and environmental factors that affect the course of the disease and can help to formulate more effective treatments guided by the real needs of individuals with this health condition [5,6]. Moreover, from a health economics perspective, information on functioning in people with schizophrenia can facilitate monitoring and help to assess the cost of disability [7], and at a macro level this information is a useful resource for public policy makers tasked with designing strategies for reducing the impact of the disease.

Previous reports have highlighted the predominant use of outcome measures that are not specifically designed for the schizophrenia population [8]. These measures prioritize the assessment of cognitive functions and psychopathology over other indicators of psychosocial disturbances and functioning such as activities and participation, and do not provide an overview of the main functioning disturbances experienced by people with schizophrenia [9,10]. In addition, instruments that assess change in functioning have low predictive validity and sensitivity and apply inadequate measurement models; this limits the use of the results obtained in contexts that require routine application to evaluate the results of interventions [11]. Achieving a holistic understanding of the situation of individuals with schizophrenia from a multidimensional and multidisciplinary assessment of their functionality and health status is crucial in order to plan specific treatments for each individual and thus improve their quality of life.

**Figure 1:**
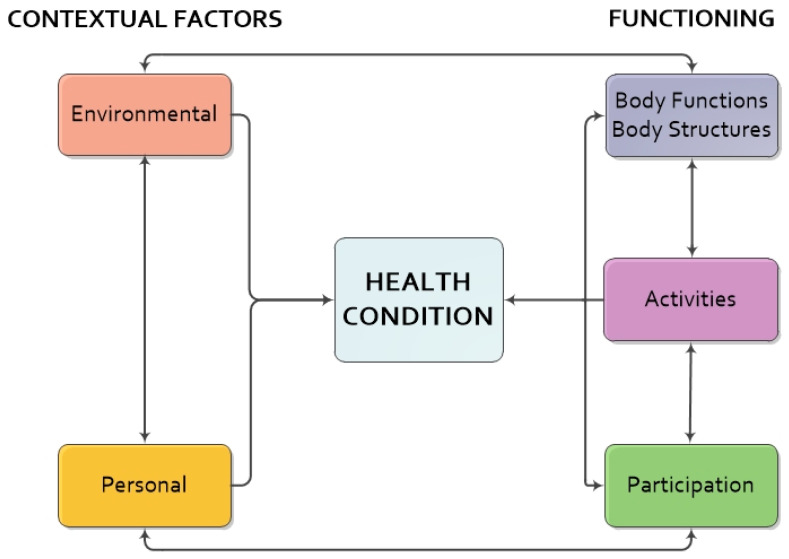
The ICF biopsychosocial model of functioning and disability. Note. Adapted from International Classification of Functioning, Disability and Health: ICF (p. 18) [12].

An approach that can help to achieve the objective of understanding the individual’s situation in a global context is the International Classification of Functioning, Disability and Health (ICF) framework. The ICF is a classification proposed and adopted in 2001 by the World Health Organization (WHO) as a comprehensive and universal measure of health and disability [12]. The ICF is based on a biopsychosocial model (see Figure 1) in which functionality and disability are conceived as a dynamic interaction between health states, composed of (a) *Body functions and Body structures*, (b) *Activities and participation*, and (c) *Contextual factors*, including *Environmental and Personal factors* [12].

Since the ICF has more than 1400 comprehensive categories applicable to different health conditions, its full implementation in daily clinical practice is challenging. It requires a large investment of time, and categories may be found that do not provide information or are not relevant in specific cases. For these reasons, it is necessary to form sets of categories that allow the description and comparison of areas of functioning for a specific health condition. These sets, called ICF Core Sets (ICF-CS), must be suitable for use in different acute, post-acute, and chronic healthcare contexts, and different health conditions [13]. Similarly, they must be adapted to different languages and cultural groups and validated through consensus with experts, people with the disease, and professionals, so as to obtain the number of categories necessary to sufficiently describe a level of functionality and health problems in the context of a particular diagnosis. So far, more than 39 ICF-CS have been developed for physical and mental diseases [13], including the ICF-CS for schizophrenia [14], which can be consulted at https://www.icfresearch-branch.org/icf-core-sets-projects2/mental-health/icf-core-setfor-schizophrenia.

The ICF-CS for schizophrenia is composed of 97 second-level categories, which are divided as follows: 17 categories from the *Body functions* component, 48 categories from the *Activities and participation* component, and finally 32 categories from *Environmental factors* component.

The ICF-CS for schizophrenia represents an important step forward because it facilitates communication and coordination in integrated care systems involving interdisciplinary teams. In addition, the ICF-CS for schizophrenia creates a unified language regarding the health phenomenon and its different dimensions, which can facilitate the comparison of results between different countries or populations. This is precisely the main objective of this study, which is carried out in the specific context of Russia. The study assesses the prevalence of functioning problems based on the ICF framework in a sample of Russian persons with schizophrenia and examines the relationship between functioning problems and socio-demographic and clinical variables.

## 2. Materials and Methods

### 2.1. Study Design

An empirical cross-sectional study was carried out in which a psychiatrist assessed the functioning of people with schizophrenia using the ICF-CS for schizophrenia. The research protocol and the consent form were approved by the ethics committees of the University of Barcelona (Spain) and the Moscow Research Institute of Psychiatry (Russian Federation).

### 2.2. Participants

Individuals over 18 years old with a primary diagnosis of schizophrenia were included. Participants were excluded if they had: 1. a primary diagnosis of another mental disorder (e.g., affective disorders, substance use disorders); 2. a serious medical or neurological pathology; 3. a surgical wound or serious injury that had not yet fully healed; 4. intellectual disability; 5. sensory disability; 6. difficulty understanding and speaking the language in which the study questions were asked; 7. significant acute positive symptomatology; 8. severe cognitive impairment. In addition, care was taken to ensure that all participants understood the research objectives, and all signed the informed consent form.

### 2.3. Instruments and Measures

Socio-demographic and clinical data of the participants were collected by means of a questionnaire. In addition, a psychiatrist assessed the subjects’ functioning and disability using the ICF-CS for schizophrenia, which contained the definitions of the categories in Russian obtained from the WHO translation. Finally, the psychiatrist assessed the subjects’ general health and functioning on two 11-point scales (one for health and one for functioning), where a score of 0 was defined as poor (poor health; poor functioning) and one of 10 as excellent (excellent health; excellent functioning).

The ICF-CS categories belonging to the *Body functions* and *Activities and participation* components were evaluated by a 5-point Likert-type scale that indicated the presence or absence of a functioning problem in that category and, if present, its severity. The grades on the scale indicated: 0, no impairment/difficulty; 1, mild impairment/difficulty; 2, moderate impairment/difficulty; 3, severe impairment/difficulty; and 4, complete impairment/difficulty in the category assessed.

The *Environmental factors* component assessed how environmental factors affected the person’s functioning, either positively or negatively. Thus, each category was evaluated both as a barrier (i.e., its negative impact) and as a facilitator (i.e., its positive impact), using a 5-point Likert scale, where 0 indicated that the category had no influence as a barrier/facilitator; 1, a mild influence as a barrier/facilitator; 2, a moderate influence; 3, a severe or substantial influence; and 4, the category was a complete barrier/complete facilitator.

### 2.4. Procedures and Data Collection

The participants were recruited at the Moscow Research Institute for Psychiatry between November 2019 and February 2020. All patients who met the inclusion criteria were contacted while they were hospitalized at that institution (they had a mean of 19.4 days of hospitalization, SD = 12.5). The purpose of the study was explained to them and they were given an informed consent form to read and sign.

The assessment of the functioning of all participants was carried out by the same psychiatrist at the institution, who had extensive knowledge of the cases. The psychiatrist made the assessment from his or her experience of the case, consulting the person’s clinical history, conducting direct observation, contacting caregivers and family members, reviewing the tests that had been administered to the person, or questioning him or her directly in a semi-structured interview. The maximum time taken for the assessment was 45 min.

### 2.5. Data Analysis

In the first instance, the socio-demographic and clinical variables of the sample and the responses in the ICF-CS categories were analyzed using descriptive statistics. To obtain the frequencies and percentages of the ICF-CS categories, responses were dichotomized as follows: categories scored between 1 and 4 were coded as “impairment”, and those as 0 as “no impairment”. Thus, the frequency and percentage of individuals showing impairment in a specific category were obtained with respect to the number of individuals assessed in that category. Only the categories that were assessed as “impairment” (scores 1 to 4) for at least 10% of the sample were considered for the analyses.

On the other hand, in order to establish the degree to which each chapter of the ICF-CS was a problem, a difficulty or a barrier and/or facilitator for the individuals, a composite score was calculated based on the 5-point Likert-type scale used to evaluate each category. This composite score consisted of adding the scores of the categories belonging to the same chapter and dividing the total obtained by the number of categories that comprised the chapter and would have been considered as an “impairment” for at least 10% of the individuals.

With this new composite score, the possible differences between groups according to the socio-demographic and clinical variables of the sample were analyzed, using the Student’s *t*-test or Mann–Whitney U test, depending on the conditions. Moreover, the composite scores of each chapter of the ICF-CS were correlated with socio-demographic and clinical variables such as age, time of illness, and number of hospitalizations due to psychotic episodes in order to assess the degree of association between each participant’s functioning assessment score and the variables already described. All data analyses were performed using SPSS 25 (IBM, Armonk, NY, USA) for Windows.

## 3. Results

### 3.1. Socio-Demographic and Clinical Characteristics

There were 40 participants (60% male), with a mean age of 37.7 years. Most of them lived with their family of origin (65%) and many of them were single (65%). They had a mean of 15 years of education (SD = 2.7), the vast majority were unemployed (75%) and were receiving a pension due to the disease (57.5%). Mean illness duration was 9.5 years (SD = 8.3), the mean number of hospitalizations due to a psychotic episode was 7.9 (SD = 6.3), and practically all (97.5%) were receiving a pharmacological treatment due to schizophrenia. Most participants were receiving a combination of two antipsychotic medications (65.0%), several were being treated with a single antipsychotic medication (27.5%), and a small proportion had been prescribed three antipsychotic drugs (5.0%). In addition, 38 participants (95.0%) were receiving another drug treatment in addition to antipsychotics (e.g., antidepressants, benzodiazepines, or other drugs to improve extrapyramidal side effects related to antipsychotic therapy). Only one participant (2.5%) was not receiving any pharmacological treatment at the time of the assessment. The socio-demographic and clinical characteristics of participants are summarized in Table 1.

### 3.2. Assessment of Functioning in Individuals with Schizophrenia

Of the 97 categories that comprise the ICF-CS for schizophrenia, 92 (94.9%) scored to some degree as a problem for the functioning of the individual in at least 10% of the sample (see Table 2, Table 3 and Table 4). In 16 of the 17 categories (94.1%) of the *Body functions* component, functioning problems were reported, the sole exception being category *b156 Perceptual functions* (chapter *b1 Mental functions*) (see Table 2).

In the case of the *Activities and participation* component, 46 of the 48 categories (95.8%) of this component were considered to be a problem for the functioning of at least 10% of the sample. Thus, only the categories *d855 Non-remunerative employment* and *d870 Economic self-sufficiency* of the *Major life areas* chapter were not a problem for any of the sample (see Table 3).

With regard to the *Environmental factors* component, only 11 of the 32 categories (34.4%) were rated to some degree as a problem in at least 10% of the cases (see Table 4). Of the 32 categories of this component, 30 (93.8%) were considered to be facilitators for functioning in at least 10% of the sample; the only exceptions were *e440 Individual attitudes of personal care providers and personal assistants* and *e555 Associations and organizational services, systems, and policies* (see Table 4).

Finally, the psychiatrist’s assessment (on a scale of 0 to 10) yielded mean scores of 5.6 (SD = 1.2) for health and 5.0 (SD = 1.5) for functioning.

When analyzing the association of socio-demographic variables with individuals’ functioning, differences were found between women and men with regards to the assessment of functioning in two chapters of the ICF-CS. Chapter *e1 Products and technology* was a greater facilitator for women (mean: 3.17, SD = 0.55) than for men (mean: 2.78, SD = 0.61), *t(38)* = 2.059, *p* < 0.001; as was chapter *e3 Support and relationships* (women’s mean score: 1.77, SD = 0.77; men’s mean score: 1.26, SD = 0.65), *t(38)* = 2.172, *p* < 0.001.

Regarding the groups of individuals who received an aid or a pension for the disease and those who did not, differences were found with respect to the assessment of functioning in the following chapters of the ICF-CS: *d1 Learning and applying knowledge* (mean ± SD: 1.50 ± 0.81 vs. 0.98 ± 0.67; *t(38*) = 2.14, *p* < 0.001, *p* = 0.039), *d4 Mobility* (median: 0.5 vs. 0.0; U = 112.5, *p* = 0.010), *d5 Self-care* (median: 0.25 vs. 0.00; U = 120.5, *p* = 0.034), *d6 Domestic Life* (median: 1.00 vs. 0.17; U = 123.5, *p* = 0.046), *d9 Community, social and civic life* (median: 0.75 vs. 0.25; U = 101.5, *p* = 0.009).

We also analyzed the correlations between the socio-demographic variables and the assessment of the subject in the different chapters of the ICF-CS, without finding significant correlations between the variables. However, when analyzing the correlations between clinical variables and the assessment of the person’s functioning, a significant correlation was found between the number of hospitalizations due to psychotic episodes and the score for chapter *d9 Community, social and civic life* (r_s_ = 0.347).

## 4. Discussion

This empirical study assesses the functioning of a sample of people with schizophrenia in a psychiatric hospital in Moscow, Russia, using the ICF-CS for schizophrenia. The sample mostly comprised male, single people living with their family of origin, who were middle-aged, and unemployed; these socio-demographic variables are common characteristics of individuals with schizophrenia [1]. For example, it has been found that in 2019, the worldwide prevalence of schizophrenia is higher in men than in women [2]; additionally, in schizophrenia, the stigma attached to the illness and its symptoms represents a major problem to finding and maintaining employment. In this regard, negative symptoms, such as blunted effect or apathy, and cognitive impairment present in the disease, become a problem for the individual at work due to them not being able to meet the demands of the workplace and therefore of maintaining a job [15]. Thus, people with the illness are often prevented from working even if they want to [9,16,17]; and are forced to give up an autonomous and independent life and live with their families of origin.

The functioning problems found in this sample are consistent with the prototypical symptoms found in the course of the illness [1]. In the case of the *Body functions* component, the most frequently reported functioning problems are mainly associated with cognitive problems (*b144 Memory functions, b140 Attention functions, b160 Thought functions* and *b164 Higher-level cognitive functions*), language (*b330 Fluency and rhythm of speech functions*), sexual functioning (*b640 Sexual functions*), weight maintenance (*b530 Weight maintenance functions*), and negative emotional symptoms (*b152 Emotional functions*), which are consistent with the problems that typically occur during the course of the disease [1,18].

A result that at first seems paradoxical is that category *b156 Perceptual functions*, from the *Mental functions chapter*, directly related to symptoms of schizophrenia such as hallucinations and delusions [1], was not considered as a category of functioning problems for this sample. The explanation is three-fold: first, the study was conducted in a clinical context (i.e., a controlled environment in a psychiatric hospital); second, the study participants were not in an active phase and no participant displayed acute positive symptoms; and third, most of the participants (97.5%) were receiving medication for their mental health condition, which controls the frequency and severity of symptoms such as hallucinations and delusions [19,20]. Another aspect that may explain this result is that most of the participants were chronic patients, and some studies have shown that personal factors, such as resilience, may explain long-term outcomes in recovery and adequate functioning in the individuals [21]. Some studies also reported an adequate level of functioning in individuals with schizophrenia without achieving complete symptomatic remission. For example, Peritogiannis and Nikolaou [22] reported that in rural settings in Greece, individuals with schizophrenia display a satisfactory level of functioning despite their partial remitted symptomatology. According to the authors, one possible explanation for this result is living in a rural environment. Rural communities may be more predisposed to help people with mental health conditions in their social relationships and in finding and keeping a job, and the individuals can function satisfactorily despite their symptoms. Klærke et al. [23] studied the long-term symptomatic and functional outcomes of unmedicated patients after a first psychotic episode and found that 19% of the long-term participants had an adequate functional remission but did not achieve symptomatic remission, showing that, in some cases, people with schizophrenia can be functional despite their symptoms.

In the *Activities and participation* component, restrictions and limitations were found in several categories with an impact on individuals’ daily functioning. Examples of these are the difficulties related to cognitive problems such as attention problems (*d160 Focusing attention*) and learning new skills (*d155 Acquiring skills*); in this regard, previous studies have already shown how limitations in these areas directly affect functioning at the occupational level [16,24,25]. Another problem that affects daily life is the stress of dealing with the symptoms of schizophrenia and the psychological overload of suffering from this mental illness. This is evident in the results, insofar as category *d240 Handling stress and other psychological demands* is the one in which most subjects show functional limitations, as well as in the category *d770 Intimate relationships.* Studies with caregivers have also stressed that the presence of physical or psychological stressors is perceived as a barrier in the recovery process of the people in their charge, especially when there is an increase in other simultaneous stressors related to the course of the disease [26]; these studies have also highlighted the frequent lack of coping skills to manage stressful situations; in particular, stress related to the positive symptoms of schizophrenia [27,28]. The last point to make with regard to this component concerns the difficulties faced by people with schizophrenia in developing intimate relationships with other people due to prejudice and discrimination [29]. This situation often leads people with schizophrenia to self-stigmatize and socially isolate themselves, thus further limiting their opportunities for generate intimate relationships [30].

In the case of the *Environmental factors* component, generally speaking, these categories are not considered a barrier to functioning in this sample: only nine of the 32 categories are seen as barriers, and only two of these nine were rated as barriers for more than 20% of the participants. The first category to be considered a barrier is *e110 Products or substances for personal consumption* (25%), which may refer, on the one hand, to the consumption of substances such as alcohol or drugs that negatively impact the cognitive functioning of individuals with schizophrenia [31], or, on the other, to the side effects of drugs and antipsychotics taken to treat the illness, which can become an impediment to functioning [32,33,34]. The second category considered as a barrier for 22.5% is *e460 Societal attitudes*, in accordance with previous studies that have shown that people judge individuals with schizophrenia in a negative way, and stigmatize and discriminate against them, very often due to their ignorance of the illness and its symptoms. This rejection may generate a chronic stressor for the individuals and create difficulties at the psychosocial level [9,35].

When *Environmental factors* are assessed as facilitators of participants functioning, 30 of its 32 categories appear as facilitators. This tendency to assess the *Environmental factors* of the Core Set more as facilitators of functioning than as barriers has also been reported in a previous study of a sample of Spanish individuals with schizophrenia [36].

Other *Environmental factors* that facilitate the functioning of these individuals are also similar to the ones identified in an earlier study [36]. For example, in the present study, the categories *e125 Products and technology for communication* and *e165 Assets* were considered as facilitators for all individuals. On the one hand, access to technology such as smartphones or computers is a fundamental advantage for people with schizophrenia as it helps them to contact people close to them and to communicate quickly with health professionals when necessary. On the other hand, having possessions and money at one’s disposal (referred to in category *e165 Assets*), has a positive impact on the participant’s functioning, given that financial autonomy helps the person to feel independent, useful, and socially capable.

In agreement with previous work [37], this study reflects the importance of health services, professionals, and family in helping patients to cope with the disease. Those individuals who perceive the support provided by others are less likely to feel unprotected and misunderstood. For example, involving families in interventions and educating them on the symptoms and characteristics of the disease can provide support for the person with the disease and reduce relapses and hospitalizations [27]. In addition, for individuals with schizophrenia, finding a balance between the treatment and the support they receive from family and health system professionals and the maintenance of their independence has a positive impact on their functioning [38,39]. For example, categories such as *e310 Immediate family* (97.5%), *e355 Health professionals* (95%), and *e580 Health services, systems, and policies* (92.5%) are seen as facilitators for a large part of the sample. This result supports that the existence of public policies and health systems that respond to the specific needs of mental illnesses such as schizophrenia may result in better treatment, better recovery rates, and lower costs associated with disability.

Regarding the relationship between socio-demographic variables and participants’ functioning, statistically significant differences according to gender were found in chapters *e1 Products and technology and e3 Support and relationships*. Technology products were a greater facilitator of functioning for women than for men, and women also obtained more support in their personal relationships than men. The fact that *e3 Support and relationships* is a greater facilitator of functioning for women in this sample may be explained by a higher prevalence of negative symptoms and disorganization in men and a higher prevalence of affective symptoms in women [40]; but also, by the cultural role of women whereby they are encouraged to seek support for coping with stressful situations and illness more often than men [41]. It would be interesting to determine whether this is the case in other contexts and cultures.

Additionally, differences were found in four chapters between individuals who received a pension for their illness and those who did not: *d1 Learning and applying knowledge*; *d4 Mobility*; *d5 Self-care*; *d6 Domestic life*; *d9 Community, social, and civic life*. Those who received financial support presented more problems of functioning. Previous studies [42] mainly propose two explanations for this situation. The first is that individuals who receive a pension for schizophrenia are possibly those who have a higher degree of disability. The second is that receiving grants or financial support from the state or family negatively impacts functioning in real life—for example, because individuals are reluctant to give up financial support in order to take on a job [42]. These findings highlight that public health policies must consider that financial support alone is not enough to positively impact a persons’ functioning and must be accompanied by interventions that respond specifically to the needs of people with schizophrenia in the chapters just mentioned.

Finally, our results show that an increase in the number of hospitalizations due to schizophrenia has a negative impact on people’s functioning in social and community life. This finding supports the impact that schizophrenia symptoms and hospitalizations can have on the social life, causing social isolation and leading to deteriorations in physical and mental health [9].

A main strength of this study is that it is the first of its kind to assess the functioning of people with schizophrenia in clinical practice using the ICF-CS for schizophrenia in Russia. This approach from the ICF framework to the clinical assessment of people with schizophrenia is relevant given the biopsychosocial nature of the illness in the Russian cultural and socio-health context. Another strength of the study is that the socio-demographic characteristics of the sample are prototypical of people with schizophrenia; the fact that the ICF-CS categories with the highest numbers of functioning problems correspond to the common symptoms of schizophrenia bears witness to the usefulness of the ICF framework in these conditions.

Despite these advantages, when applying the ICF-CS it is important to consider the objective to be achieved, because although it provides us with comprehensive information about functioning, it can be very time-consuming to apply. Applying the ICF-CS can be very useful when the aim is to work in rehabilitation programmes in an interdisciplinary way, when an in-depth description of the person’s functioning is needed, or when a person is in long-term care and it is required to go deeper into his or her functioning [43]. If the aim is to screen the person’s functioning as a first approach to his or her situation in the daily clinical routine, the use of the brief ICF-CS would be better, which is composed of the minimum number of categories needed to adequately describe and assess the functioning and disability of the person with a specific health condition. In the case of the brief ICF-CS for schizophrenia, its component categories can be found on the ICF Research Branch Centre website (https://www.icf-research-branch.org/download/category/9-mentalhealth)—accessible to anyone who may need to use it.

The study also has certain limitations. First, this research was carried out with a population recruited at a single psychiatric hospital and in a particular city in a particular country, a circumstance that limits the generalizability of the results obtained. Indeed, the results, particularly those concerning the *Environmental factors* component of the ICF-CS may be affected by social, cultural, and economic variables specific to the context of Moscow and Russia, where the research was carried out. Therefore, further studies in an international context are needed to corroborate the results, and care must be taken when trying to generalize the findings. Second, the participants were people with schizophrenia who did not have severe cognitive impairment, so when extrapolating the results and conclusions of the study, this characteristic of the sample must be considered. A final limitation has to do with the study sample, which, on the one hand, is of limited size and, on the other hand, is a convenience sample recruited in the Russian context, which may affect the representativeness of the data and their potential extrapolation.

In future studies, a wider range of health professionals should assess the functioning of people with schizophrenia using the ICF-CS. This is because previous research on the content validity of the ICF-CS with different professionals (nurses, occupational therapists, physiotherapists, psychiatrists, psychologists, and social workers) has shown that the difference in perspective between health professionals means that some ICF-CS categories for schizophrenia may be more relevant than others for a given health professional, although the way of assessing functionality is the same. [44,45,46,47,48,49].

In addition, further research could continue this study by expanding it beyond the specific context in which it was carried out. Russia is a very large country, with a population of 146.7 million inhabitants in 2019 [2] and with a high degree of cultural variability. A first step in advancing the assessment of the functioning of people with schizophrenia in the country with the ICF framework would be to apply the ICF-CS in different centres and regions throughout this culturally diverse country.

## 5. Conclusions

Functioning problems in schizophrenia are highly prevalent in the Russian context, and several relevant areas related to significant daily activities are affected. The assessment of functioning from the ICF framework, which is based on a biopsychosocial and integrative perspective, may provide a more concrete understanding of the functioning and disability of the person with schizophrenia and its relationship to their context, which may allow for situation-specific treatment planning that has better outcomes and a positive impact on people’s lives and functioning.

Using the ICF-CS for schizophrenia to assess the functioning of individuals may promote new public health policies, in this case in the Russian context, that respond to the real problems of functioning of people with schizophrenia.

## Figures and Tables

**Table 1:** Socio-demographic and clinical variables of participants (*n* = 40).

Variables	*n* (%)	Mean (SD)
Gender		
Male	24 (60.0)	
Female	16 (40.0)	
Patient’s living environment		
Rural	5 (12.5)	
Urban	35 (87.5)	
Living arrangements		
Family of origin	26 (65.0)	
Own family	4 (10.0)	
Alone	7 (17.5)	
Other	3 (7.5)	
Marital status		
Single	26 (65.0)	
Married/cohabiting	4 (10.0)	
Separated/Divorced	9 (22.5)	
Widowed	1 (2.5)	
Current primary occupation		
Student	3 (7.5)	
Self-employed	2 (5.0)	
Paid employment	5 (12.5)	
Unemployed	30 (75.0)	
Receiving pension		
No	17 (42.5)	
Yes	23 (57.5)	
Is the patient receiving pharmacological treatment due to schizophrenia?		
Yes	39 (97.5)	
No	1 (2.5)	
Age (years)		37.7 (12.7)
Formal education (years)		15.0 (2.7)
Illness duration (years)		9.5 (8.3)
Number of hospitalizations due to a psychotic episode during lifetime		7.9 (6.3)
General health (0 to 10 scale)		5.6 (1.2)
General functioning (0 to 10 scale)		5.0 (1.5)

**Table 2:** Frequency and percentage of participants with impairments * on the ICF categories of the *Body functions* component.

Chapter	ICF Code	ICF Category Name	Frequency (%)
1. Mental functions	b114	Orientation (time, place, person)	6 (15.0)
	b117	Intellectual functions	12 (30.0)
	b122	Global psychosocial functions	11 (27.5)
	b130	Energy and drive functions	16 (40.0)
	b134	Sleep functions	13 (32.5)
	b140	Attention functions	28 (70.0)
	b144	Memory functions	30 (75.0)
	b147	Psychomotor functions	15 (37.5)
	b152	Emotional functions	22 (55.0)
	b156	Perceptual functions	3 (7.5) **
	b160	Thought functions	23 (57.5)
	b164	Higher-level cognitive functions	19 (47.5)
	b180	Experience of self and time functions	11 (27.5)
3. Voice and speech functions	b330	Fluency and rhythm of speech functions	25 (62.5)
5. Functions of the digestive, metabolic and endocrine systems	b530	Weight maintenance functions	23 (57.5)
6. Genitourinary and reproductive systems	b640	Sexual functions ^1^	22 (61.11)
7. Neuromusculoskeletal and movement-related functions	b765	Involuntary movement functions	15 (37.5)

* Impairment means that the category has a score between 1–4, ** This category is a problem for less than 10% of the sample and is therefore removed from the analyses, ^1^ Sample size *n* = 36: data was missing for four people in this category.

**Table 3:** Frequency and percentage of participants with impairment * on the ICF categories of the *Activities and participation* component.

Chapter	ICF Code	ICF Category Name	Frequency (%)
1. Learning and applying knowledge	d155	Acquiring skills	22 (55.0)
	d160	Focusing attention	29 (72.5)
	d163	Thinking	18 (45.0)
	d166	Reading	19 (47.5)
	d175	Solving problems	15 (37.5)
	d177	Making decisions	22 (55.0)
2. General tasks and demands	d210	Undertaking a single task	6 (15.0)
	d220	Undertaking multiple tasks	20 (50.0)
	d230	Carrying out daily routine	15 (37.5)
	d240	Handling stress and other psychological demands	33 (82.5)
3. Communication	d310	Communicating with—receiving—spoken messages	14 (35.0)
	d315	Communicating with—receiving—nonverbal messages	8 (20.0)
	d330	Speaking	22 (55.0)
	d335	Producing nonverbal messages	14 (35.0)
	d350	Conversation	19 (47.5)
4. Mobility	d470	Using transportation	11 (27.5)
	d475	Driving ^1^	7 (21.2)
5. Self-care	d510	Washing oneself	4 (10.0)
	d520	Caring for body parts	8 (20.0)
	d540	Dressing	8 (20.0)
	d570	Looking after one’s health	15 (37.5)
6. Domestic life	d610	Acquiring a place to live	11 (27.5)
	d620	Acquisition of goods and services	6 (15.0)
	d630	Preparing meals	19 (47.5)
	d640	Doing housework	19 (47.5)
	d650	Caring for household objects	5 (12.5)
	d660	Assisting others	18 (45.0)
7. Interpersonal interactions and relationships	d710	Basic interpersonal interactions	8 (20.0)
	d720	Complex interpersonal interactions	12 (30.0)
	d730	Relating with strangers	6 (15.0)
	d740	Formal relationships	9 (22.5)
	d750	Informal social relationships	17 (42.5)
	d760	Family relationships ^2^	18 (46.1)
	d770	Intimate relationships ^3^	23 (79.3)
8. Major life areas	d820	School education	16 (40.0)
	d825	Vocational training ^4^	11 (28.2)
	d830	Higher education ^5^	13 (43.3)
	d840	Apprenticeship (work preparation) ^6^	12 (30.7)
	d845	Acquiring, keeping, and terminating a job	20 (50.0)
	d850	Remunerative employment	21 (52.5)
	d855	Non-remunerative employment	3 (7.5) **
	d860	Basic economic transactions	4 (10.0)
	d865	Complex economic transactions	8 (20.0)
	d870	Economic self-sufficiency	1 (2.5) **
9. Community, social, and civic life	d910	Community life ^7^	4 (13.8)
	d920	Recreation and leisure	22 (55.0)
	d930	Religion and spirituality ^8^	9 (23.7)
	d950	Political life and citizenship ^9^	18 (46.1)

* Impairment means that the category has a score between 1–4, ** This category is a problem for less than 10% of the sample and is therefore removed from the analyses. ^1^ Sample size *n* = 33: data was missing for seven people in this category. ^2,4,6,9^ Sample size *n* = 39: data was missing for one person in these categories. ^3,7^ Sample size *n* = 29: data was missing for eleven people in these categories. ^5^ Sample size *n* = 30: data was missing for ten people in this category. ^8^ Sample size *n* = 38: data was missing for two people in this category.

**Table 4:** Frequency and percentage of participants with environmental barriers and/or facilitators * on the ICF categories of the *Environmental factors* component.

Chapter	ICF Code	ICF Category Name	Barriers	Facilitators
			Frequency (%)	Frequency (%)
1. Products and technology	e110	Products or substances for personal consumption	10 (25.0)	39 (97.5)
	e125	Products and technology for communication	5 (12.5)	40 (100.0)
	e130	Products and technology for education	4 (10.0)	31 (77.5)
	e165	Assets	2 (5.0) **	40 (100.0)
3. Support and relationships	e310	Immediate family	8 (20.0)	39 (97.5)
	e315	Extended family	2 (5.0) **	21 (52.5)
	e320	Friends	2 (5.0) **	21 (52.5)
	e325	Acquaintances, peers, colleagues, neighbours and community members	4 (10.0)	20 (50.0)
	e330	People in positions of authority ^1^	0 (0.0)	11 (28.2)
	e340	Personal care providers and personal assistants	1 (2.5) **	6 (15.0)
	e355	Health professionals	5 (12.5)	38 (95.0)
	e360	Other professionals	2 (5.0) **	20 (50.0)
4. Attitudes	e410	Individual attitudes of immediate family members	1 (2.5) **	17 (42.5)
	e415	Individual attitudes of extended family members	1 (2.5) **	7 (17.5)
	e420	Individual attitudes of friends	3 (7.5) **	8 (20.0)
	e425	Individual attitudes of acquaintances, peers, colleagues, neighbours and community members	2 (5.0) **	6 (15.0)
	e430	Individual attitudes of people in positions of authority ^2^	0 (0.0)	9 (23.08)
	e440	Individual attitudes of personal care providers and personal assistants	0 (0.0)	3 (7.5) **
	e450	Individual attitudes of health professionals	1 (2.5) **	26 (65.0)
	e455	Individual attitudes of other professionals	2 (5.0) **	15 (37.5)
	e460	Societal attitudes	9 (22.5)	20 (50.0)
	e465	Social norms, practices, and ideologies	1 (2.5) **	36 (90.0)
5. Services, systems, and policies	e525	Housing services, systems, and policies	5 (12.5)	27 (67.5)
	e545	Civil protection services, systems, and policies	6 (15.0)	32 (80.0)
	e550	Legal services, systems, and policies	7 (17.5)	16 (40.0)
	e555	Associations and organizational services, systems, and policies ^3^	0 (0.0)	2 (5.1) **
	e560	Media services, systems, and policies	8 (20.0)	28 (70.0)
	e570	Social security services, systems, and policies	0 (0.0)	26 (65.0)
	e575	General social support services, systems, and policies	0 (0.0)	22 (55.0)
	e580	Health services, systems, and policies	0 (0.0)	37 (92.5)
	e585	Education and training services, systems, and policies	1 (2.5) **	29 (72.5)
	e590	Labour and employment services, systems, and policies	1 (2.5) **	8 (20.0)

* Barrier or facilitator means that the category has a score between 1–4, ** This category is a problem for less than 10% of the sample and is therefore removed from the analyses. ^1–3^ Sample size *n* = 39: data was missing for one person in these categories.

## Data Availability

The data presented in this study are available on request from the last author.
